# Supplementary material for: Fusarium graminearum‐induced shoot elongation and root reduction in maize seedlings correlate with later seedling blight severity
Source: Plant Direct. 2018 Jul 18;2(7):e00075. doi: 10.1002/pld3.75 (PMC6508817; doi:10.1002/pld3.75)
Supplement: Supplementary file 2 [file PLD3-2-e00075-s002.pdf]

Reviewer #1:

In this study, the responses (root and shoot length) of 9 commercial maize hybrids and 15 inbred lines to three Fusarium graminearum isolates (Gz014, Gz835, Gz829) were evaluated. In general, F. graminearum infection induced shoot elongation and root reduction in maize seedlings.

Comments:
In the abstract and through the paper, the authors described shoot elongation and root reduction as morphological changes. I am not sure that this is an adequate term as no histological or other anatomical description were performed. Maybe "effects on growth and elongation" would be more accurate.

>>We now refer to shoot elongation and root reduction as “growth response.”

One of the main conclusions from this study is that the fungal isolate provides the main source of assay variance. This means that the biology of each isolate is key in the development of seedling blight. In this sense, how do these three isolates differ in deoxynivalenol production? What additional information is available on these fungal isolates regarding their virulence?

>>As mentioned in text (line 103-106), these three isolates were chosen from a previous isolate aggressiveness test (Kuhnem et al., 2015). They produce the same dominant deoxynivalenol.

I suggest moving Tables 1, 2 and 3 to supplemental materials.

>>Changes have been made as suggested.

Fig. 1. Delete "+/- s.d." from the axis label. It is indicated in the legend.

>>Bar plots have been changed to box-and-whisker plots as suggested by the editor.

Fig. 2. Delete "+/- s.d." from the axis label. It is indicated in the legend. Fig. 2B shows representative photos of mock-inoculated and inoculated roots. Indeed, the full root system is less-developed in the inoculated plant, but the main root seems to be longer in inoculated plants than on mock plants. Is this true? Did the authors collect data on this parameter? How can it be explained?

>>Length data of primary root were not specifically collected, and hence it is not possible to determine if the longer main root observed by the reviewer was statistically significant.

Fig. 3. I suggest modifying the Y-axis legend to A. Ratio of Root Length / B. Ratio of Shoot Length. And indicate in the legend that the ratio refers to Fungus/Mock-Inoculated. Please include minor marks in the Y-axis.

>>This figure has been changed to show both the uninfected and infected root lengths, rather than the ratio.

Fig. 4. I suggest modifying the Y-axis legend to A. Ratio of Root Length / B. Ratio of Shoot Length. And indicate in the legend that the ratio refers to Fungus/Mock-Inoculated. Please include minor marks in the Y-axis.

>>We have changed the Y-axis legend as suggested

Fig. 5 A/B. I suggest modifying the Y-axis legend to A. Ratio of Root Length / B. Ratio of Shoot Length. And indicate in the legend that the ratio refers to Fungus/Mock-Inoculated. Please include minor marks in the Y-axis.

>>Changes have been made as suggested.

Fig. 6. Title legend could be: F. graminearum-induced growth parameter changes in roots and shoots, and seedling blight severity, which are significantly correlated with each other.

>>Changes have been made as suggested.

Editor's comments and suggestions:

- If as the authors discuss the typical phenotype in the field is stunted growth, can the authors use the lab conditions system to predict line resistance in the field?

>>To the best of our knowledge, this experiment has not been done. To answer the reviewer’s question, quantitative assessment of maize seedling blight severity following controlled inoculation under field conditions is required. While this question is relevant and probably could be answered building on results presented here, it is beyond the scope of the current study.

- line 77. Is not clear how the works presented above justifies the idea that Gibberella stalk rot and F. graminearum seedling blight are resistance are controlled by similar genetic and physiological mechanisms.

>>We hypothesize that Gibberella stalk rot and *F. graminearum* seedling blight resistance may have similar genetic and physiological mechanisms because near-isogenic lines bred to have increased Gibberella stalk rot resistance also showed lower *F. graminearum* seedling blight severity. The sentence in text has been rephrased for clarification.

- line 115. You mean that the seedlings where 24h old since germination?

>>Seedlings were inoculated when their primary roots reached approximately 9 cm, and the exact days that took varied by maize genotype. The sentence in text has been updated for clarification.

- Fig 1 legend. Please, be more specific about the lines that were used in this experiment. They are not mentioned in the legend.

>>Changes have been made as suggested.

- Fig 2 and 3 legends. Please, specify the concentration of spores that

was used here?

>>Changes have been made as suggested.

- Fig 6. It would be useful to draw regression lines for each fungal isolate.

>>Since we concluded that the correlative relationships were primarily driven by differences in fungal isolates, we think isolate-specific regression lines on the figure would add more confusion than information.

- Finally, I suggest considering using alternatives to bar plots. Check the reasons here:
http://journals.plos.org/plosbiology/article?id=10.1371%2Fjournal.pbio.1002128
http://thenode.biologists.com/barbarplots/photo/

>>Bar plots have been changed to box-and-whisker plots, and the figure legends have been updated. We prefer leaving Figure 4 as a bar plot to allow easier comparison between the different treatment groups.
